# Effect of Supplemental Bamboo Leaf Extract on Milk Production, Composition, Biochemical Indices, and Fecal Microbiota Diversity in Grazing *Yili* Mares

**DOI:** 10.3390/life15121928

**Published:** 2025-12-17

**Authors:** Chuankun Wang, Jianwen Wang, Bingqiang Ma, Ting Liu, Xinxin Yuan, Jun Meng, Yaqi Zeng

**Affiliations:** 1 College of Animal Science and Technology, Xinjiang Agricultural Vocational and Technical University, Changji 831100, China; wck94214@163.com; 2 Xinjiang Key Laboratory of Horse Breeding and Exercise Physiology, College of Animal Science and Technology, Xinjiang Agricultural University, No. 311 Nongda East Road, Urumqi 830052, China

**Keywords:** *Yili* horses, plant flavonoid, milk production, microbiota

## Abstract

**Purpose**: This study investigated the effects of dietary bamboo leaf extract (BLE) on milk parameters and intestinal microbiota in lactating *Yili* mares. **Methods**: Twenty-four *Yili* mares of similar age (10 ± 2 years), weight (360.62 ± 15.23 kg) and body condition were selected for this study and randomly divided into four groups of six mares each: an untreated control group (CG) and three experimental groups (EG1, EG2, EG3) were fed a basal diet supplemented with 0, 10, 20, or 30 g/day of BLE, respectively, for 60 days. Then, horse milk composition, antioxidant activity, and immunoglobulin levels along with the relative abundance of fecal microbiota were measured. **Results**: Compared with the control group, supplementation with BLE for 60 days significantly improved milk yield and composition. The protein content in the EG1 was significantly higher than that in the CG, the milk yield and fat content in the EG2 was significantly higher than that in the CG, and the lactose content in the EG3 was significantly higher than that in the CG. BLE also significantly increased the milk’s antioxidant capacity, vitamin C, IgG, IgM, and IgA levels, with the antioxidant and immune properties in the EG2 being significantly higher than those in the CG. Furthermore, BLE feeding promoted communities of beneficial intestinal microbes. Bacteria associated with energy metabolism and organic matter decomposition increased significantly in BLE-fed groups, especially the EG2, which had elevated abundance of *UCG-002* and the *NK4A214_group*. BLE also significantly reduced the abundance of Euryarchaeota, Verrucomicrobiota, *Methanobacteriaceae*, and *Methanobrevibacter*. **Conclusions**: Dietary supplementation with bamboo leaf extract is a safe and inexpensive way to enhance milk yield and quality and to promote the growth of beneficial intestinal microbes in *Yili* horses.

## 1. Introduction

Bamboo leaf extract (BLE) is a plant flavonoid preparation with rich biological activity that is used as a natural feed additive in livestock and poultry production. Regarding the effect of dietary BLE on milk constituents, it has been reported to increase lactation and the content of milk protein and lactose in cows [1,2], but few studies have reported its use in horses. In addition, supplemental feeding of BLE had a positive effect on antioxidant capacity (MDA, T-AOC, Vitamin C) and immune function (serum IgM, IL-1β, TNFα), yet different feeding cycles, concentration, and dosage may lead to variable results [3].

Lactation requires a large amount of energy. The difference between the minimum and maximum energy intake during lactation in animals can be as high as 1.9-fold [4], and a mare’s need for energy may be greater under grazing conditions [5]. As the organ responsible for the process of digestion, the intestinal tract is also important in its capacity to act as a barrier against pathogenic bacteria, thus playing a pivotal role in immunity. The gut contains a rich community of microbes that aid in digestion and produce a variety of nutrients, such as amino acids, fatty acids, vitamins, and peptides. These microbes play a crucial role in the regulation of host metabolism, energy production, and the immune response [6,7]. Horses are classified as hindgut fermenters due to the presence of active communities of fiber-degrading bacteria and fungi in their hindgut. This intestinal flora is capable of fermenting complex carbohydrates from fiber-rich feeds into volatile fatty acids (VFAs), which account for 60–70% of the horse’s daily energy requirements [8]. Therefore, stabilizing the gut microbial structure is critical for lactating mares. BLE has good antioxidant activity and strong antibacterial effects [9], and a moderate intake helps to maintain homeostasis in animals [10]. Analysis of the fecal microbiota of animals directly reflects the changes in the internal microbial environment of the gastrointestinal tract [11]. On this basis, it was deemed feasible to measure the effect of BLE on the composition of the intestinal microbiota by determining the fecal microbial diversity of the mares.

To our knowledge, the effect of BLE supplementation has been little studied in horses compared to other livestock species such as goats and cows. Previous research demonstrated that supplemental feeding of BLE improved milk secretion, during lactation, and increased its milk production in *Yili* horses [12,13]. However, the potential links between milk components and fecal microorganisms have yet to be investigated. Therefore, the aim of this study was to investigate the effects of supplemental feeding of different levels of BLE on milk yield, composition, and biochemical indices, and the fecal microbiota of *Yili* horses. Also, the correlation between fecal microorganisms and milk composition was also studied in order to provide a rational basis for the addition of BLE to their diet.

## 2. Materials and Methods

### 2.1. Bamboo Leaf Extracts

The Bamboo Leaf Extracts selected for this study was purchased from Shaanxi Senyuan Biotechnology Co., Ltd. which comes from Xi’an City, Shaanxi Province, China. The raw material for bamboo leaf extract is primarily made from dried bamboo (*Lophatherum gracile Brongn*, *Gramineae*) stems and leaves. The production method involves sun-drying, cutting into segments, and pulverizing. The active ingredients in the Bamboo were extracted and concentrated into a brownish-yellow powder with a particle size of 80–100 mesh. The flavonoid content of the BLE was 60%, mainly consisting of flavonoids, lactones, and phenolic acids [14].

### 2.2. Animal Selection and Experimental Design

The experiment was conducted from July to September at the Alibek Breeding Station, Zhaosu Horse Farm, *Yili* Kazakh Autonomous Prefecture, Xinjiang Uygur Autonomous Region, at an altitude of 1000–1200 m, with average temperatures of 11 °C in July, 9 °C in August, and 7 °C in September. All procedures in this study were approved by the Animal Experiment Ethics Committee of Xinjiang Agricultural University (permit No.: 2018012).

In this study, 24 Yili mares were randomly selected and divided into 4 groups of 6. The mean age of the mares was 10 ± 2 years and their mean weight was 360.62 ± 15.23 kg. All mares underwent veterinary health examinations prior to the experiment to ensure they were in good physical condition and healthy. A free-range management system was employed throughout the experiment. The horses were fed 1 kg of corn (Table 1) per day along with BLE at 0 g/d (CG), 10 g/d (EG1), 20 g/d (EG2) and 30 g/d (EG3) for 7 days as a pre-test, and then for 60 days as the experimental cycle. The 7-day pre-experiment primarily involves desensitizing mares through milking and fecal sample collection.

### 2.3. Feeding and Management

All horses were kept in the same rearing environment during the test. They were fed a corn-based diet and given tap water ad libitum. Horses were herded to the paddock each morning at 7:30 a.m. They were directed to different feeding stations at 8 a.m. The BLE was mixed with the crushed corn in the feed pocket for feeding each day. After the feeding, the mares were transferred to the paddock. Milk production measurements were taken every 7 days. During the measurement period, foals were kept separate from their mares. After the measurement period concluded, foals and mares were reunited. External markings were applied to the mare’s body prior to the experiment, and samples were collected in the same sequence each time.

### 2.4. Collection of Samples

#### 2.4.1. Milk Sampling

Manual milking was performed every 2 h (10:00, 12:00, 14:00, and 16:00), and the milk yield of each mare from the four milking sessions (8 h, 8:00 a.m. to 16:00 p.m.) was calculated. Milk production was recorded every 7 days. Horse milk samples were collected every 14 days, specifically at 0 days, 14 days, 28 days, and 56 days, using 100 mL sterile tubes (Beijing Compson Biotechnology Co., Ltd. Beijing, China), and stored at −20 °C for later analysis.

#### 2.4.2. Fecal Sampling

Fecal samples were collected by rectal sampling into 5 mL sterile tubes on days 0, 30, and 60. Three tubes of fresh fecal samples were collected per mare, and stored at −20 °C until analysis. On Day 60, one mare each from the CG group and EG1 group received veterinary deworming treatment; so, fecal samples were not collected. Therefore, a total of 70 samples was used for 16S-RNA sequencing analysis.

### 2.5. Analysis of Samples

#### 2.5.1. Milk Production

Milk yield was measured using a micrometer electronic scale (Shenhua Biotechnology Co., Ltd., Hangzhou, China). Four measurements were made each time and the data were combined to estimate the total daily (24 h) milk yield.

#### 2.5.2. Milk Composition, Antioxidant Capacity, and Immune Indicators

Milk composition was determined using an automated analyzer (Lactoscan MCC50 automatic milk analyzer, Nova Zagora, Bulgaria). Antioxidant levels (MDA, T-AOC, SOD, CAT GSH-PX, Vitamin C) and immune response markers (IgG, IgM, IgA) in the milk were measured by colorimetric methods (Myriad BS-420 automatic biochemical analyzer, Shenzhen Mindray Bio-Medical Electronics Co., Ltd., Shenzhen, China. and DR-200BS ELISA reader, Wuxi Huawede Lang Instrument Co., Ltd., Wuxi City, China.).

#### 2.5.3. Fecal Microbiota

Genomic DNA was extracted from fecal samples and amplified by PCR using 16S rRNA primers [15]. Amplicons were purified, subjected to library construction, and sequenced on an Illumina Nova platform.

### 2.6. Data Analysis

Excel 2019 was used for initial data organization. Each indicator was statistically represented by data with repeated observations using the Mixed Model in SAS 9.2 software. The fixed effects included Trt (treatment), date, and Trt × Date. The treatment group is the main factor of the analysis and discussion. The variance structure was determined using an AR model, the data were expressed as least squares mean ± standard error (SE), and multiple comparisons were performed using the Pdiff method, with *p* < 0.05 considered statistically significant and *p* < 0.01 considered highly significant. Correlation analyses were performed using the Pearson modules in SPSS (version 19.0), with *p* < 0.05 and *p* < 0.01 considered significant and highly significant, respectively.

## 3. Results and Analysis

### 3.1. Effects of BLE on Milk Production and Composition

**Figure 1:**
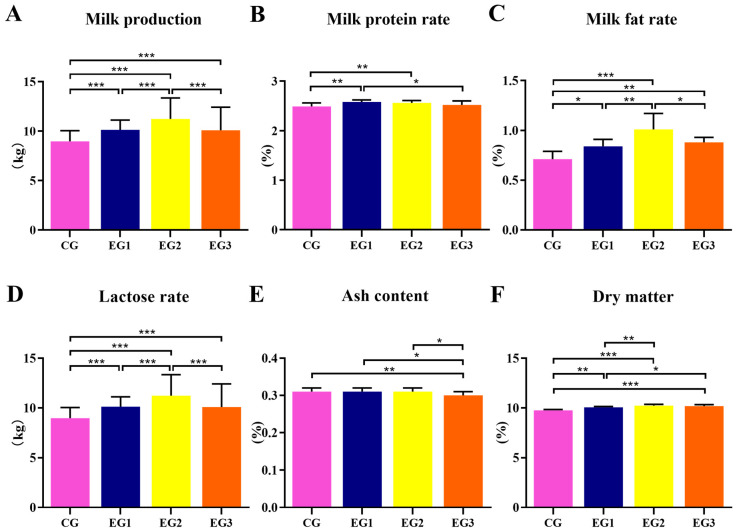
Effect of supplemental feeding of BLE on milk production and composition in lactating *Yili* horses. (**A**) Milk production; (**B**) Milk protein rate; (**C**) Milk fat rate; (**D**) Lactose rate; (**E**) Ash content (**F**) Dry matter. Notes: *** *p* < 0.001, ** *p* < 0.01, * *p* < 0.05. CG = control group; EG1 = experiment group I; EG2 = experiment group II, EG3 = experiment group III.

Figure 1A shows that dietary supplementation with BLE (0–60 d) affected milk composition compared with the control group (CG), and the milk yield was elevated in EG1, EG2, and EG3 (*p* < 0.01). Compared with the CG, the milk protein rate (Figure 1B) was elevated in EG1 and EG2 (*p* < 0.01); the milk fat rate (Figure 1C) and lactose rate (Figure 1D) were increased in EG1 (*p* < 0.05), EG2 (*p* < 0.01), and EG3 (*p* < 0.01); and the dry matter content (Figure 1F) was elevated in EG1 (*p* < 0.01), EG2 (*p* < 0.01), and EG3 (*p* < 0.01). Compared with EG3, the ash content (Figure 1E) was lower in the CG (*p* < 0.01) (see Supplementary Table S1 for detailed data).

### 3.2. Effects of BLE on Antioxidant Capacity in Milk

**Figure 2:**
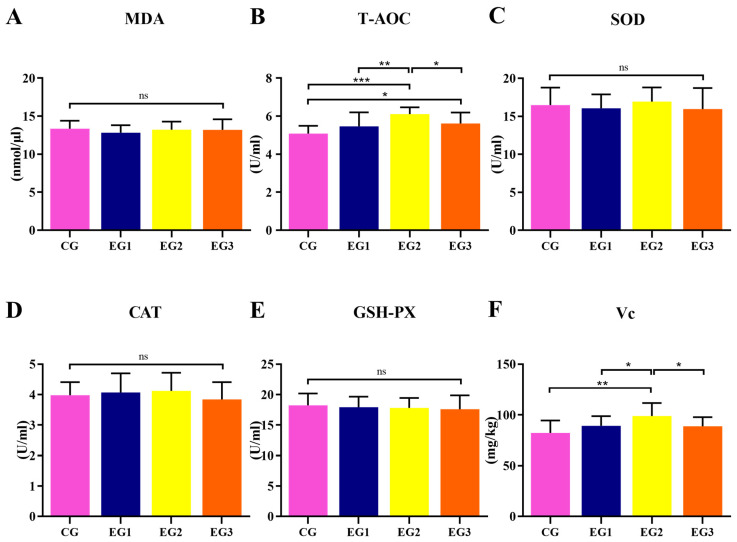
Effect of supplemental feeding of BLE on antioxidant indices in the milk of lactating *Yili* horses. (**A**) MDA; (**B**) T-AOC; (**C**) SOD; (**D**) CAT; (**E**) GSH-PX (**F**) Vc. Notes: *** *p* < 0.001, ** *p* < 0.01, * *p* < 0.05, ns *p* > 0.05.

As shown in Figure 2, compared with the CG, the T-AOC was elevated in EG2 (*p* < 0.01) and EG3 (*p* < 0.05), and Vitamin C content was elevated in EG2 (*p* < 0.01) (see Supplementary Table S2 for detailed data).

### 3.3. Effect of BLE on Immune Response Markers in Milk

**Figure 3:**
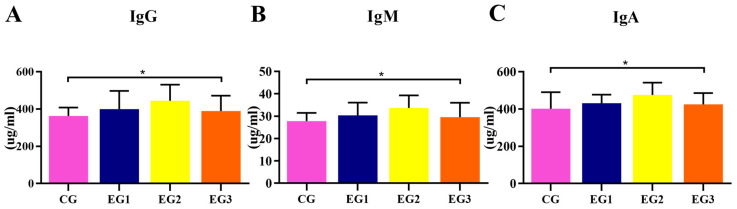
Effect of supplemental feeding of BLE on immunological indices in the milk of lactating *Yili* horses. (**A**) IgG; (**B**) IgM; (**C**) IgA Notes: * *p* < 0.05.

As shown in Figure 3, compared with the CG, the IgG, IgM, and IgA contents were increased in the experimental groups, and the elevation was highest in EG2 (*p* < 0.05) (see Supplementary Table S3 for detailed data).

### 3.4. Effects of BLE on Fecal Microbial Diversity

**Figure 4:**
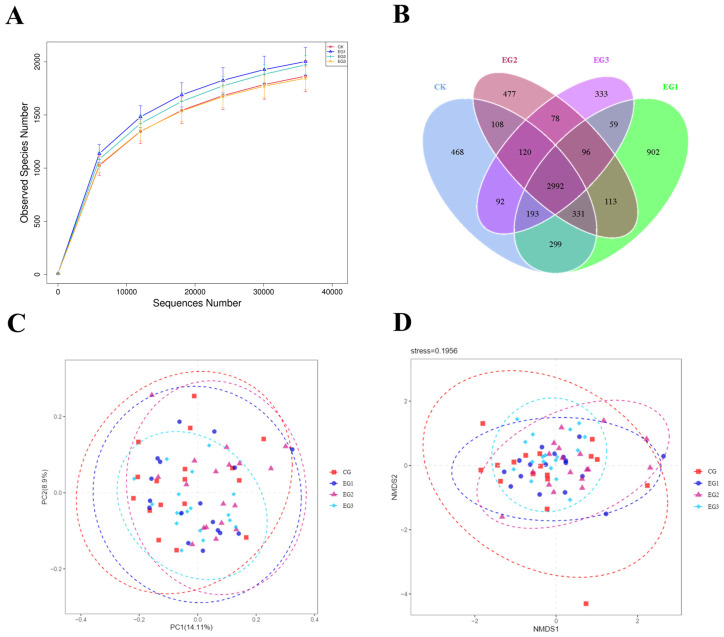
(**A**) Number of valid sequences in each of the 0–60 d groups; (**B**) Venn plots of OTU sequences; (**C**) NMDS analysis; (**D**) PCA analysis.

**Figure 5:**
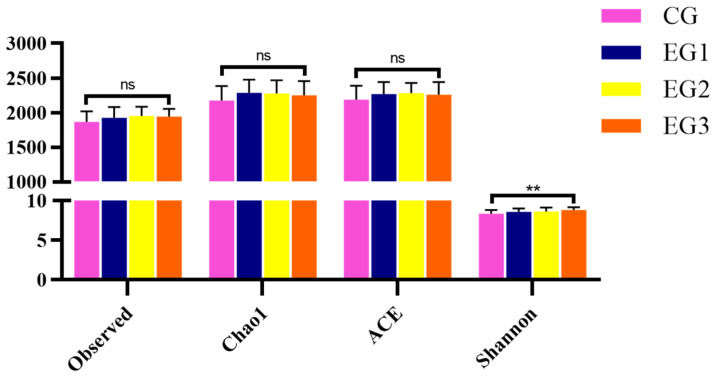
Microbial α-diversity in fecal microbiota of *Yili* horses. Notes: ** *p* < 0.01, ns *p* > 0.05.

The sequencing coverage of the intestinal microbiota of *Yili* horses was all above 98.5% (Figure 4A). The number of Operational Taxonomic Unit (OTU) sequences is shown in Figure 4B, and the effect of aggregation of intestinal microbiota became more evident with increasing BLE feeding (Figure 4C,D). There were inter-group differences in the Shannon index of α-diversity in the fecal microbiota, with EG3 higher than in the CG (*p* < 0.01) (Figure 5) (see Supplementary Table S4 for detailed data).

### 3.5. Effect of BLE on Abundance of Fecal Microbes at the Phylum, Family, and Genus Level

**Figure 6:**
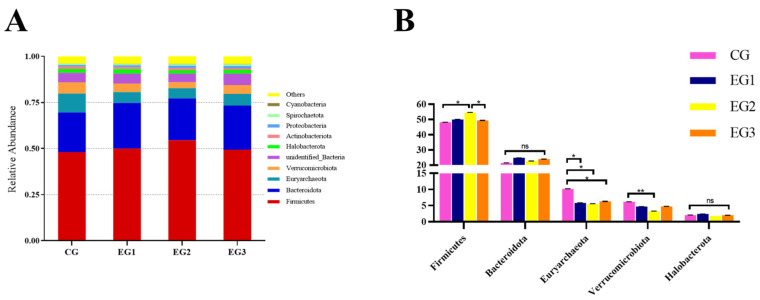
Relative abundance of fecal microbiota at the phylum level in *Yili* horses. (**A**) The abundance of fecal microbes at the phylum Level; (**B**) The results at the phylum level in CG, EG1, EG2 and EG3. Notes: ** *p* < 0.01, * *p* < 0.05, ns *p* > 0.05.

The results at the phylum level (Figure 6B) showed that the abundance of Firmicutes in EG2 was higher than in the CG and EG3 (*p* < 0.05); the abundance of Euryarchaeota was higher in the CG than in EG1, EG2, or EG3 (*p* < 0.05); and the abundance of Verrucomicrobiota in the CG was higher than in EG2 (*p* < 0.01) (see Supplementary Table S5 for detailed data).

**Figure 7:**
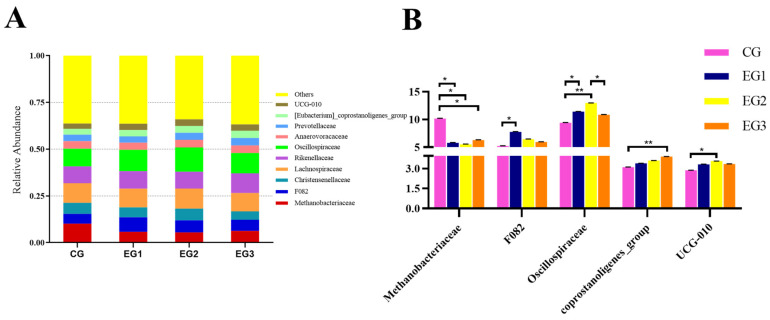
Relative abundance of fecal microbiota at the family level in *Yili* horses. (**A**) The abundance of fecal microbes at the family Level; (**B**) The results at the family level in CG, EG1, EG2 and EG3. Notes: ** *p* < 0.01, * *p* < 0.05.

The results at the family level (Figure 7B) showed that the abundance of *Methanobacteriaceae* in the CG was higher than that in EG1, EG2, or EG3 (*p* < 0.05); the abundance of *F082* in EG1 was higher than in the CG (*p* < 0.05); the abundance of *Oscillospiraceae* in EG2 was higher than in the CG (*p* < 0.01) and EG3 (*p* < 0.05); the abundance of *Oscillospiraceae* in EG1 was higher than in the CG (*p* < 0.01) (see Supplementary Table S6 for detailed data).

**Figure 8:**
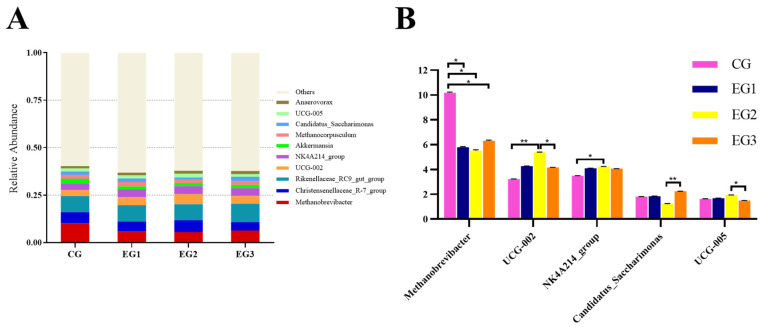
Relative abundance of fecal microbiota at the genus level in *Yili* horses. (**A**) The abundance of fecal microbes at the genus Level; (**B**) The results at the genus level in CG, EG1, EG2 and EG3. Notes: ** *p* < 0.01, * *p* < 0.05.

Among the top ten most abundant fecal microbial genera, Methanobrevibacter, Christensenellaceae_R-7_group, Rikenellaceae_RC9_gut_group, UCG-002, NK4A214_group, Akkermansia, Methanocorpusculum, Candidatus saccharimonas, UCG-005, and Anaerovorax were dominant (Figure 8A).

The results at the genus level (Figure 8B) showed that the abundance of *Methanobrevibacter* in the CG was higher than that in EG1, EG2 or EG3 (*p* < 0.05); the abundance of *UCG-002* in EG2 was higher than in the CG (*p* < 0.01) and in EG3 (*p* < 0.05). The abundance of the *NK4A214_group* in EG2 was higher than in the CG (*p* < 0.05). The abundance of *Candidatus saccharimonas* in EG3 was higher than in EG2 (*p* < 0.05), and the abundance of *UCG-005* in EG3 was lower than in EG2 (*p* < 0.05) (see Supplementary Table S7 for detailed data).

### 3.6. Correlation Between the Fecal Microbiota and BLE Concentration in Feed

**Figure 9:**
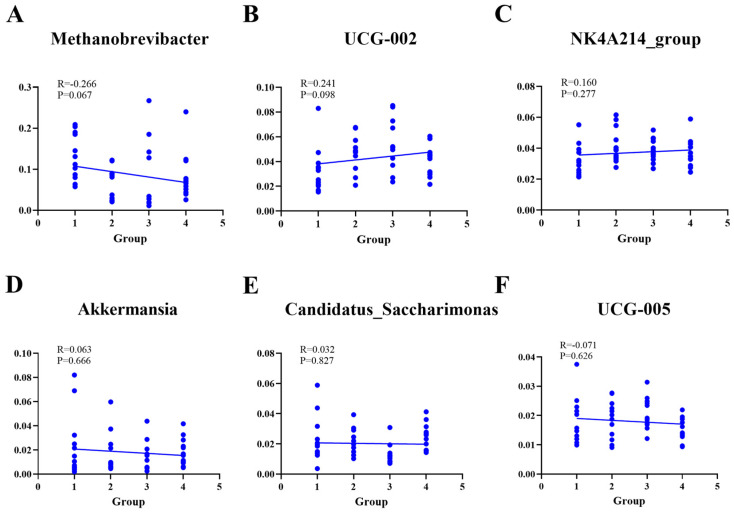
Correlation between fecal microbiota at the genus level and BLE supplementation concentration. (**A**) *Methanobrevibacter*; (**B**) *UCG-002*; (**C**) *NK4A214*_group; (**D**) *Akkermansia*; (**E**) *Candidatus_Saccharimonas*; (**F**) *UCG-005*. Notes: the numbers (1, 2, 3, and 4) on the X scale represent CG, EG1, EG2, EG3, and the 5 does not represent any group.

As shown in Figure 9A–F *UCG-002* and *NK4A214*_group were positively correlated with BLE concentration (*p* > 0.05). *Methanobrevibacter*, *Akkermansia*, and *UCG-005* were negatively correlated with BLE concentration.

### 3.7. Correlation Between Fecal Microbiota and Milk Composition

**Figure 10:**
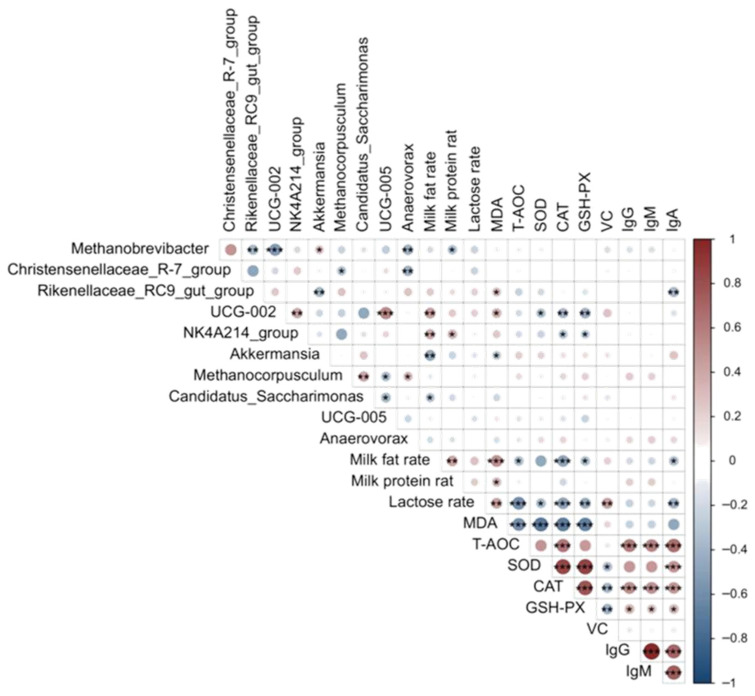
Correlation between fecal microbiota composition at the genus level and the content of specific milk components. Notes: *** *p* < 0.001, ** *p* < 0.01, * *p* < 0.05.

The correlation between the horizontal abundance of microbial genera in the feces of *Yili* horses and the content of constituents in mare’s milk is shown in Figure 10. The milk composition results showed that *UCG-002* abundance was positively correlated with the percent of milk fat (*p* < 0.01). *NK4A214_group* abundance was positively correlated with the percent of milk fat (*p* < 0.01) and milk protein (*p* < 0.05), whereas *Akkermansia* abundance was negatively correlated with percent milk protein (*p* < 0.01). *Methanobrevibacter* abundance was also negatively correlated with milk protein percentage (*p* < 0.05).

The milk antioxidant results showed that *UCG-002* abundance was positively correlated with MDA level (*p* < 0.05) and negatively correlated with SOD (*p* < 0.05), CAT (*p* < 0.01) and GSH-PX (*p* < 0.01). The *Rikenellaceae_RC9_gut_group* abundance was positively correlated with MDA (*p* < 0.05). The *NK4A214_group* abundance was negatively correlated with CAT (*p* < 0.05) and GSH-PX (*p* < 0.05). The *Akkermansia* abundance was negatively correlated with MDA content (*p* < 0.05).

The immune response results in the milk show that the relative numbers of the *Rikenellaceae_RC9_gut_group* were positively correlated with IgA (*p* < 0.01).

### 3.8. Linear Discriminant Analysis Effect Size (LEfSe)

**Figure 11:**
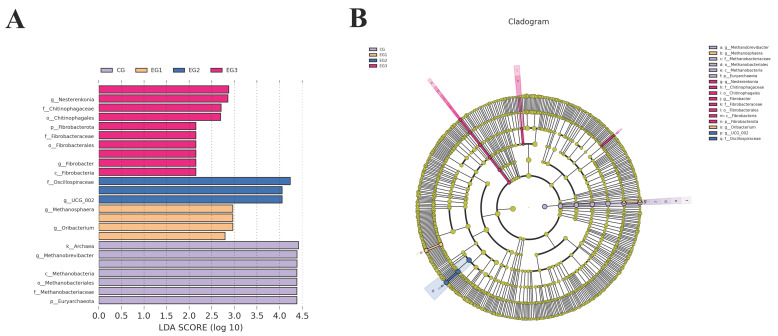
(**A**) Histogram of LDA value distribution; (**B**) Diagram of evolutionary bifurcation.

As shown in Figure 11A,B, an LDA score of 4 was used as a threshold in the LEfSe analysis. Compared to other experimental groups, the bacterial communities that were significantly different in the CG were Archaea, *Methanobrevibacter*, Euryarchaeota, *Methanobacteriaceae*, *Methanobacteria*, and Methanobacteriales. The taxa enriched in EG2 with significant differences were *Oscillospiraceae*, *UCG_002*.

### 3.9. Functional Prediction (Tax4Fun)

**Figure 12:**
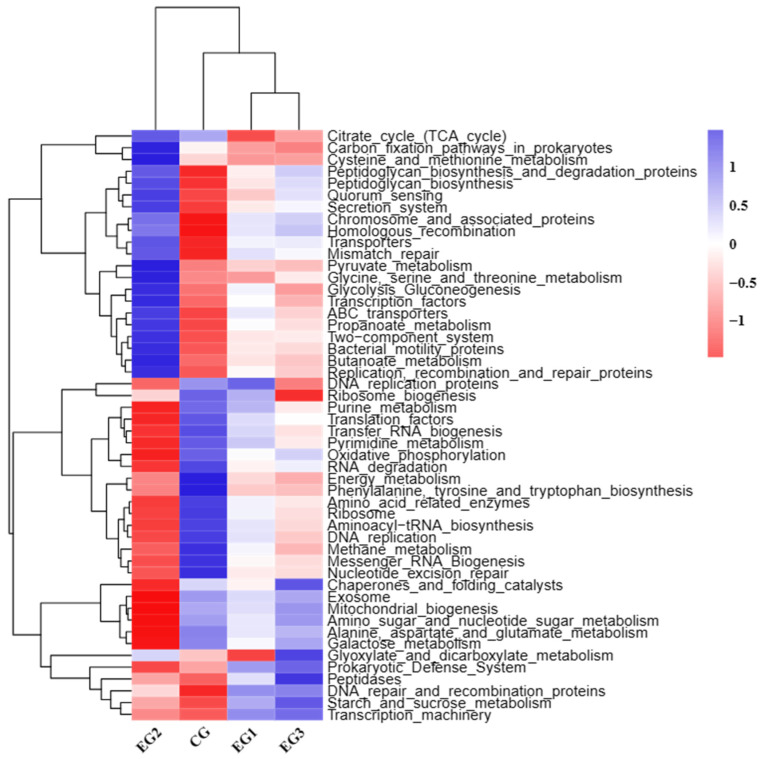
Tax4Fun analysis at level 3.

Functional analysis of the fecal microbiota in the various groups is shown in Figure 12. Fecal microorganisms in the CG were mainly involved in replication and repair, glycan biosynthesis and metabolism, membrane transport, and cellular community-prokaryotes. Fecal microorganisms in EG2 were mainly involved in the metabolism of carbohydrates, nucleotides, and energy, and protein translation, folding, sorting, and degradation. Fecal microorganisms in EG1 were primarily involved in carbohydrate metabolism and translation in EG3.

## 4. Discussion

### 4.1. Effect of BLE on Milk Production and Percent Composition

Some plant compounds such as extracts of *Pueraria candollei* [16], Gypsophila Vaccaria [17], and soy flavonoids [18] have been shown to enhance milk production in mammals. The underlying mechanisms for these lactation-enhancing effects may involve modulation of the secretion of anterior pituitary hormones [19]. Several authors have tested a range of BLE concentrations as a feed supplement for dairy cows, and the results showed that milk production was elevated but the differences between groups were not significant [2,20]. In contrast, Yi et al. used BLE to treat heat-stressed cows and obtained more definitive results, with milk production in the treated group being significantly higher than that in the control group [21]. The milk production results in this study were generally in agreement with previous data, showing that supplemental feeding of BLE significantly increased milk production in mares compared with the control group. The largest increase in milk yield was observed in the EG2 group (20 g/d), indicating that moderate supplemental feeding of BLE was particularly effective in increasing milk yield.

Supplemental feeding of soy flavonoids to increase milk protein, fat, and lactose content in dairy cows has been reported [22,23,24]. This similar result has also been reported in studies on bamboo leaf flavonoids. Mingyue et al. demonstrated that supplemental feeding with BLE increased protein and lactose in cow’s milk, and the best results were obtained with 30 g/d. The results of this experiment were consistent with those of previous studies, showing that supplemental feeding of BLE could significantly increase the percentage of milk protein, fat, and lactose, whereas the largest increase in milk protein was observed in EG1 (10 g/d), the greatest increase in milk fat in EG2 (20 g/d), and the highest lactose level occurred in EG3 (30 g/d). The increase in milk protein content in equine milk may be a result of bamboo leaf flavonoids that promote the secretion of estrogen in mares, which causes an elevation in nucleosomal RNA and increases the transcription and translation of casein mRNA, thus affecting milk protein content [23]. In addition, a study on cow’s milk showed that supplemental feeding with BLE affected the microbiota in cow’s milk, which, in turn, led to changes in milk protein levels. Moreover, supplementation with BLE significantly upregulated glycerophospholipids and fatty acyl groups in milk [20]. Ruminal acetate and propionate, precursors of milk fat synthesis, were also significantly increased [25].

### 4.2. Effect of BLE on Antioxidant Capacity

Flavonoids in plant extracts play a protective role in biological systems through their antioxidant activity [26]. The concentration of flavonoids in plasma is very low, but there is a transient increase in plasma T-AOC level after supplemental feeding of flavonoid-containing supplements; yet, most of them are rapidly metabolized in the body, reducing their antioxidant capacity [27]. Flavonoids in BLE have antioxidant effects [28], and they have been reported to reduce the content of malondialdehyde (MDA) and improve the total antioxidant capacity (T-AOC) of organisms [29,30]. The results of this study showed that supplemental feeding with BLE reduced the MDA content in mare’s milk in all experimental groups compared with the control group, but there were no significant differences among the experimental groups, which may be related to BLE concentration. Furthermore, it has been shown that the addition of BLE to feed increased the T-AOC levels in blood [14]. Our results also showed that supplemental feeding with BLE in EG2 (20 g/d) produced the greatest increase in T-AOC in mare’s milk, and the T-AOC levels in all BLE-treated groups were higher than those of the control group, consistent with previous reports [12,14]. Vitamin C is a water-soluble compound that is a natural antioxidant [31]. The vitamin C content in milk was increased in all experimental groups, but the best results were obtained in EG2 (20 g/d), which was higher than in the CG, EG1 (10 g/d) and EG3 (30 g/d). These results show that moderate supplementation of BLE can reduce MDA content and increase total antioxidant capacity (T-AOC) and Vitamin C content in mares’ milk, which, in turn, enhances antioxidant performance.

### 4.3. Effect of BLE on the Immune Response

Immunoglobulins are important components of immunoreactivity in milk, with IgG, IgM and IgA being the major immunoglobulin classes in mammary secretions. It has been hypothesized that the elevated immunoglobulin content in milk may be attributed to the estrogen-like functional effects of bamboo leaf flavonoids, which have a significant regulatory effect on immunity and promote immune protein synthesis and secretion [32]. The results of previous studies have proved that soy flavonoids could improve humoral immune function of the bovine mammary gland [33]. In this study, the levels of IgG, IgM and IgA in mare’s milk in the experimental group were significantly higher compared with CG, with the best effect in EG2 (20 g/d). It has been shown that supplemental feeding with BLE enhances the gene expression of IL-2 and IFN-γ in the spleen and promotes the secretion of IFN-γ and IL-2 in blood [34]. This may be related to the elevation in immunoproteins in milk. It is known that Glycerophospholipids play an important role in the regulation of host inflammation and immunity [35]. Likewise, sphingolipids have been shown to be involved in innate immunity against intestinal infection by pathogenic microbes [36]. The glycerophospholipids and sphingolipids are also related to the anabolism of milk components, thus confirming the anabolic effect of BLE and supporting the conclusion that supplemental feeding with BLE has an immunomodulatory effect on the milk of mares in this study.

### 4.4. Effect of BLE on Fecal Microbial Diversity

Alpha-diversity analysis reflects the richness and diversity of microbial communities within a sample. Shu Gang et al. reported that dietary addition of bamboo leaf flavonoids had a small effect on cecal microbiota diversity in broilers, but increased microbiota [35]. In this study, compared with the control group, supplemental feeding of BLE reduced the number of bacterial species in the gut microbiota of grazing *Yili* mares, but showed no significant influence on the abundance or diversity of bacterial colonies. Notably, the EG3 group exhibited a significantly higher aroma intensity index than the control group. Statistical analysis of intergroup differences in bacterial genera revealed significant increases in certain key bacterial communities, such as *UCG-002* and *NK4A214_group*, thus confirming the anabolic effect of BLE and providing further evidence to support supplemental feeding with bamboo leaf flavonoid extract.

### 4.5. Effect of BLE on Fecal Microbial Structure

*Firmicutes* and *Bacteroidota* are dominant phyla in the gut of many kinds of animals and are functionally important for host health [37,38,39,40]. It has been shown that *Firmicutes* and *Bacteroidota* account for 79–86% of the total intestinal microbiota in herbivores [41,42]. The gut microbiota of healthy horses consist of 50.49% *Firmicutes* and 30.73% *Bacteroidota* [43]. Similar results were noted in grazing *Yili* horses, with *Firmicutes*’ abundance ranging from 48.12% to 54.51% and *Bacteroidota*’s abundance ranging from 21.46% to 22.69% across groups. Furthermore, the results of intergroup comparisons in this study showed that supplemental feeding with BLE increased the abundance of *Firmicutes* and decreased the abundance of *Euryarchaeota* and *Verrucomicrobiota*; the compositional changes in the intestinal microbiota were most obvious in EG2 (20 g/d), which had significantly higher levels of *Firmicutes* than CG and EG3 (30 g/d) and significantly lower levels of *Euryarchaeota* and *Verrucomicrobiota* than CG. Studies have confirmed that *Firmicutes* are closely associated with fiber degradation [34,35], and promote the production of volatile fatty acids [8]. This study revealed through correlation analysis that supplementation with BLE significantly increased the abundance of the *UCG-002* and *NK4A214_group* microbial communities in mares. Notably, the abundance of *UCG-002* and *NK4A214_group* bacteria positively correlates with milk fat content in mares. This suggests that *UCG-002* and *NK4A214_group* may participate in cellulose degradation metabolism and promote fatty acid synthesis.

The phylum *Euryarchaeota* contains the currently known *methanogenic* bacteria. The lower methane concentrations observed in horses compared with ruminants may be attributable to the fact that ruminants release these gases through the rumen, whereas excessive methane accumulation can damage the intestine in equines [44]. The results of this experiment were similar to those obtained by Jing Lv, where supplemental feeding with BLE reduced the abundance of *Methanobacteriaceae* and *Methanobrevibacter* in lactating mares. The abundance of *Methanobacteriaceae* and *Methanobrevibacter* was significantly reduced in the experimental groups compared with CG, with the lowest abundance observed in EG2 (20 g/d). The greater abundance of *Methanobacteriaceae* and *Methanobrevibacter* in CG may be related to supplemental basal ration (cornmeal) [45], whereas their abundances decreased rather than increased in the BLE groups, which, in turn, proved that BLE had an inhibitory effect on *Methanobacteriaceae* and *Methanobrevibacter* in the intestine.

The *Oscillospiraceae* belongs to the *Firmicutes* phylum, and the type of genus of this family is *Oscillospira*. Studies confirm that the *Oscillospiraceae* are the dominant family in free-ranging animals and have the potential to synthesize butyric acid and degrade benzoic acid [46,47]. In this experiment, the abundance of *Oscillospiraceae* in the intestinal tract of horses in the experimental groups was higher than that in CG, with the highest level occurring in EG2 (20 g/d). A number of studies have demonstrated that supplemental feeding with active substances can increase the abundance of *Oscillospiraceae* in animals [48,49]. This suggests that *Oscillospiraceae* may respond positively to certain active constituent(s) of BLE that is beneficial in moderate amounts.

In mammals, the nutritional requirements are increased during gestation and lactation [50]. *UCG-002* and *NK4A214_group* are important genera of intestinal bacteria that are closely related to energy metabolism. In this investigation, supplemental feeding with BLE increased the relative abundance of *UCG-002 and NK4A214_group* in mares in the experimental group, with the highest abundance observed in EG2 (20 g/d). Interestingly, milk yield and composition also increased in the experimental groups, with the highest milk yield and milk fat percentage observed in EG2. Previous research by Cao et al. reported that dietary supplementation with BLF induced an increase in the cecal acetate and butyrate contents [14]. Therefore, it was hypothesized that *UCG-002* and *NK4A214_group* may be associated with changes in milk composition, and correlation analyses revealed a significant positive correlation between these genera and milk fat. Evidence indicates that *NK4A214_group* and *NK3A20* promoted starch and sucrose metabolism [51] and were associated with butyric acid synthesis [52,53], while short-chain fatty acids such as butyric acid are precursors for milk fat synthesis. In summary, *UCG-002* and *NK4A214_group* may be related to milk fat synthesis.

### 4.6. Prediction of Fecal Microbial Function

The results of LEfSe analysis revealed that the bacterial taxa significantly enriched in CG were mainly from Euryarchaeota, *Methanobacteria, Methanobacteriaceae*, and the genus *Methanobrevibacter*, which are mainly associated with the fermentation of organic matter and energy production [54]. The taxa significantly enriched in EG2 (20 g/d) were *Oscillospirales* and *UCG-002*, which have been associated with the regulation of lipid metabolism in the body, the promotion of lipid-lowering effects, and weight loss [47,48]. The results of functional prediction showed that the changes in the intestinal microbiota of grazing *Yili* mares supplemented with BLE were closely related to genetic information processing, metabolism, and cellular processes, and that the linkages between them need to be further explored.

## 5. Conclusions

In summary, the results of milk production, milk fat percentage, total antioxidant capacity, and immune indices of the *Yili* Mares supplemented with 20 g/d of bamboo leaf extract showed the greatest effects. Supplemental feeding with BLE increased the relative abundance of beneficial intestinal bacteria (*UCG-002*, *NK4A214_group*) in *Yili* mares, and these bacteria may be associated with an increase in milk fat content of horses. The limitations of this study are related to the design as the test animals selected were under grazing conditions; therefore, the results provide only a baseline reference and supporting data for the supplemental feeding of BLE to grazing mares.

## Figures and Tables

**Table 1:** Composition of corn meal.

Term	Percentage
Raw material composition (%)	
Cornmeal	100
nutrient composition (%)	
DM%	86
CP%	7.9
EE%	3.6
CF%	2.3
NFE%	71.8
Ash%	1.2
NDF%	9.9
ADF%	3.1
Faecula%	63.5
Ca%	0.02
P%	0.27

## Data Availability

The sequencing data that support the findings of this study are available from NCBI databases, and the BioProject number PRJNA1068315.

## Supplementary Materials

The following supporting information can be downloaded at: https://www.mdpi.com/article/10.3390/life15121928/s1, Table S1. Effect of different levels of bamboo leaf extract on milk yield and composition in lactating *Yili* horses; Table S2. Effect of different levels of bamboo leaf extract on antioxidant indices in the milk of lactating *Yili* horses; Table S3. Effect of different levels of bamboo leaf extract on immunoglobulins in the milk of lactating *Yili* horses; Table S4. Alpha diversity; Table S5. Relative abundance (%) of fecal microbiota by phylum; Table S6. Relative abundance (%) of fecal microbiota by family; Table S7. Relative abundance (%) of fecal microbiota by genus.
