# Supplementary material for: Effect of Supplemental Bamboo Leaf Extract on Milk Production, Composition, Biochemical Indices, and Fecal Microbiota Diversity in Grazing Yili Mares
Source: Life (Basel). 2025 Dec 17;15(12):1928. doi: 10.3390/life15121928 (PMC12735208; doi:10.3390/life15121928)
Supplement: Supplementary file 1 [file life-15-01928-s001.zip › life-3904021-supplementary.pdf]

Supplementary Materials

Table S1. Effect of different levels of bamboo leaf extract on milk yield and composition in lactating *Yili* horses.

Item	Control group	Trial group I	Trial group II	Trial group III	SE	*P*-value		
						Trt	Date	Trt×Date
Milk yield (kg)	8.96^{C}	10.13^{B}	11.23^{A}	10.08^{B}	0.244	<0.001	<0.001	0.892
Milk protein (%)	2.49^{Bc}	2.58^{Aa}	2.56^{Aab}	2.52^{ABbc}	0.019	0.004	0.033	0.065
Milk fat (%)	0.71^{Cc}	0.84^{BCb}	1.01^{Aa}	0.88^{ABb}	0.038	<0.001	<0.001	0.004
Lactose (%)	6.25^{Cc}	6.31^{BCb}	6.34^{Bb}	6.49^{Aa}	0.019	<0.001	<0.001	<0.001
Ash content (%)	0.31^{Aa}	0.31^{ABa}	0.31^{ABa}	0.30^{Bb}	0.003	0.011	<0.001	0.031
Dry matter (%)	9.75^{Cc}	10.05^{Bb}	10.22^{Aa}	10.18^{ABa}	0.041	<0.001	<0.001	<0.001

Trt: trial treatment; Date: sampling time point. Values in the same row with the same lowercase letter or without a superscript letter are not significantly different ($P>0.05$), while values with different lowercase letters are significantly different ($P<0.05$). Values with different uppercase letters are extremely significantly different ($P<0.01$). The same notation is used in the following tables.

Table S2. Effect of different levels of bamboo leaf extract on antioxidant indices in the milk of lactating *Yili* horses.

Item	Control group	Trial group I	Trial group II	Trial group III	SEM	*P*-value		
						Trt	Date	Trt×Date
MDA (nmol/μl)	13.35	12.81	13.21	13.20	0.294	0.608	<0.001	0.227
T-AOC (U/ml)	5.08^{Bc}	5.46^{Bbc}	6.11^{Aa}	5.61^{ABb}	0.158	<0.001	<0.001	0.039
SOD (U/ml)	16.48	16.05	16.93	15.95	0.628	0.605	0.032	0.415
CAT (U/ml)	3.98	4.07	4.12	3.84	0.168	0.656	0.028	0.279
GSH-PX (U/ml)	18.22	17.93	17.80	17.58	0.564	0.875	0.034	0.400
Vc (mg/kg)	82.13^{Bb}	89.19^{ABb}	98.90^{Aa}	88.77^{ABb}	3.267	0.008	0.110	0.087

Table S3. Effect of different levels of bamboo leaf extract on immunoglobulins in the milk of lactating *Yili* horses.

Item	Control group	Trial group I	Trial group II	Trial group III	SEM	*P*-value		
						Trt	Date	Trt×Date
IgG (μg/ml)	363.38^{b}	399.34^{ab}	444.96^{a}	389.48^{ab}	22.522	0.094	<0.001	0.377
IgM (μg/ml)	27.80^{b}	30.37^{ab}	33.72^{a}	29.56^{ab}	1.560	0.071	<0.001	0.574
IgA (μg/ml)	401.84^{b}	431.04^{ab}	475.33^{a}	425.41^{ab}	19.276	0.071	0.022	0.820

Table S4. Alpha diversity.

Item	Control group	Trial group I	Trial group II	Trial group III	SE	*P*-value		
						trt	date	trt×date
Goods (%)	98.9	98.8	98.8	98.8	0.99	0.664	<0.001	0.044
Observed	1865.03	1925.79	1951	1940.28	31.98	0.232	0.007	0.732
Chao1	2172.29	2283.42	2278.12	2249.78	2084.66	0.257	0.006	0.075
ACE	2188.81	2269.46	2278.44	2259.35	2109.19	0.369	0.008	0.292
Shannon	8.36^{B}	8.57^{AB}	8.61^{AB}	8.8^{A}	0.11	0.044	0.21	0.627
Simpson	0.99	0.99	0.99	0.99	0.002	0.185	0.147	0.278

Table S5. Relative abundance (%) of fecal microbiota by phylum.

Item	Control group	Trial group I	Trial group II	Trial group III	SE	*P*-value		
						trt	date	trt×date
Firmicutes	48.12^{b}	49.97^{ab}	54.51^{a}	49.36^{b}	1.72	0.051	0.057	0.411
Bacteroidota	21.46	24.79	22.69	23.97	1.54	0.439	0.008	0.48
Euryarchaeota	10.17^{a}	5.81^{b}	5.51^{b}	6.31^{b}	1.3	0.047	<0.001	0.237
Verrucomicrobiota	6.17^{A}	4.68^{AB}	3.27^{B}	4.75^{AB}	0.66	0.026	0.001	0.994
Unidentified_Bacteria	5.15^{ABb}	5.37^{ABab}	4.73^{Bab}	6.27^{Aa}	0.03	0.017	0.017	0.54
Halobacterota	2.05	2.39	1.75	2.02	0.42	0.744	0.358	0.791
Actinobacteriota	1.37	1.12	1.1	0.85	0.21	0.403	0.002	0.954
Proteobacteria	0.83	0.97	1.34	1.25	0.25	0.449	0.093	0.741
Spirochaetota	0.64	0.65	0.84	0.97	0.16	0.397	0.086	0.816
Cyanobacteria	0.12	0.15	0.16	0.16	0.04	0.826	0.725	0.208
Others	3.92	4.08	4.1	4.08	0.17	0.86	<0.001	0.964

Table S6. Relative abundance (%) of fecal microbiota by family.

Item	Control group	Trial group I	Trial group II	Trial group III	SE	*P*-value		
						trt	date	trt×date
Methanobacteriaceae	10.17^{a}	5.81^{b}	5.51^{b}	6.31^{b}	1.3	0.047	<0.001	0.237
F082	5.23^{b}	7.78^{a}	6.45^{ab}	5.96^{ab}	0.88	0.224	0.343	0.929
Christensenellaceae	5.89	5.29	6.26	4.48	0.66	0.232	0.309	0.936
Lachnospiraceae	10.51	10.05	10.74	9.84	0.73	0.799	<0.001	0.044
Rikenellaceae	9.01	9.31	8.99	10.51	0.76	0.425	<0.001	0.795
Oscillospiraceae	9.43^{Bcd}	11.41^{ABab}	12.97^{Aa}	10.86^{ABbc}	0.69	0.006	0.202	0.801
Anaerovoracaceae	4.03	3.82	4.06	4.02	0.19	0.817	<0.001	0.222
Prevotellaceae	3.45	3.38	3.76	3.98	0.37	0.641	0.003	0.306
[Eubacterium]_coprostanoligenes_group	3.11^{B}	3.39^{AB}	3.60^{AB}	3.90^{A}	0.21	0.060	0.076	0.663
UCG-010	2.87^{b}	3.32^{ab}	3.56^{a}	3.35^{ab}	0.18	0.069	0.575	0.367
Others	36.29	36.43	34.11	36.79	1.09	0.281	0.043	0.903

Table S7. Relative abundance (%) of fecal microbiota by genus.

Item	Control group	Trial group I	Trial group II	Trial group III	SE	*P*-value		
						trt	date	trt×date
Methanobrevibacter	10.17[a]	5.79[b]	5.51[b]	6.31[b]	1.30	0.047	<0.001	0.237
*Christensenellaceae*_R-7_group	5.87	5.28	6.22	4.47	0.65	0.238	0.306	0.932
*Rikenellaceae*_RC9_gut_group	8.43	8.59	8.44	9.64	0.70	0.542	<0.001	0.881
UCG-002	3.22[Bb]	4.26[ABab]	5.38[Aa]	4.15[ABb]	0.41	0.005	0.165	0.938
NK4A214_group	3.49[b]	4.09[ab]	4.23[a]	4.06[ab]	0.25	0.164	0.013	0.344
Akkermansia	2.39	1.53	1.54	1.74	0.40	0.379	0.004	0.704
Methanocorpusculum	2.05	2.39	1.75	2.02	0.42	0.745	0.359	0.791
Candidatus saccharimonas	1.80[AB]	1.84[AB]	1.24[B]	2.24[A]	0.23	0.030	0.007	0.404
UCG-005	1.63[ab]	1.68[ab]	1.94[a]	1.49[b]	0.15	0.179	0.085	0.360
Anaerovorax	1.23	1.39	1.54	1.58	0.12	0.171	<0.001	0.490
Others	59.71	63.15	62.20	62.29	1.45	0.383	<0.001	0.185
